# Respiratory Sinus Arrhythmia Moderates the Relation between Parent-Adolescent Relationship Quality and Adolescents’ Social Adjustment

**DOI:** 10.1007/s10802-015-9989-7

**Published:** 2015-02-26

**Authors:** Jolien Van der Graaff, Wim Meeus, Minet de Wied, Anton van Boxtel, Pol van Lier, Susan Branje

**Affiliations:** 1 Research Centre Adolescent Development, Utrecht University, PO Box 80.140, 3508 TC Utrecht, The Netherlands; 2 Department of Psychology, Tilburg University, PO Box 90.153, 5000 LE Tilburg, The Netherlands; 3 Department of Developmental Psychology, VU University Amsterdam, 1081 BT Amsterdam, The Netherlands

**Keywords:** Resting RSA, Empathy, Externalizing behavior, Parenting, Adolescence

## Abstract

This 2-wave longitudinal study aimed (1) to investigate whether high resting RSA predicted adolescents’ lower externalizing behavior and higher empathic concern, and (2) to address the potential moderating role of resting RSA in the association between parent-adolescent relationship quality and adolescents’ externalizing behavior and empathic concern. In a sample of 379 adolescents (212 boys, 167 girls), resting RSA was assessed during a laboratory session, and adolescents reported on parental support, negative interaction with parents, empathic concern and externalizing behavior during a home visit. We found no support for high resting RSA predicting low externalizing behavior or high empathic concern. However, in line with our hypotheses, we did find several instances of RSA functioning as a moderator, although the interaction patterns varied. First, negative interaction with parents was a negative predictor of externalizing behavior for girls low in resting RSA, whereas the association was non-significant for girls with high RSA. Second, higher negative interaction with parents predicted lower empathic concern for boys high in resting RSA, whereas the association was reversed for boys with low resting RSA. Third, parental support was a positive predictor of empathic concern for girls high in resting RSA, whereas the association was non-significant for girls low in resting RSA. The findings suggest that adolescents with different levels of resting RSA respond differentially to relationship quality with parents.

Self-regulation is assumed to play an important role in adolescents’ social functioning. High self-regulation has been related to lower levels of externalizing behavior and to higher levels of empathic concern (e.g., Fabes et al. 1994; Oldehinkel et al. 2007). Resting respiratory sinus arrhythmia (RSA) is generally seen as a physiological marker of dispositional self-regulation (Porges 1991; Thayer and Lane 2000). Individuals low in resting RSA, indicating reduced parasympathetic activation of the heart, are thought to have more difficulties in physiological and behavioral self-regulation and are therefore less capable of displaying situation-appropriate reactions than individuals high in resting RSA (Porges 1991). Low resting RSA indeed has been related to higher externalizing behavior in clinical or at risk samples (see Kibler et al. 2004) and to lower empathic concern (e.g., Fabes et al. 1993). Besides potential direct effects, there are conceptual reasons to expect resting RSA to moderate the association between environmental influences and adolescents’ adjustment, although theories regarding the nature of this interaction are diverging (see Eisenberg et al. 2012). Therefore, we examined in a community sample of adolescent boys and girls (1) whether low resting RSA predicted higher externalizing behavior, (2) whether low resting RSA predicted lower empathic concern, and (3) whether and how resting RSA moderated the link between parent-adolescent relationship quality and adolescents’ social functioning.

## Resting Respiratory Sinus Arrhythmia (RSA) as a Marker of Self-Regulation

RSA is the high frequency component of heart rate variability, and is a measure of the magnitude of the rhythmic fluctuations in heart rate across the respiratory cycle which are characterized by increasing heart rate during inhalation and decreasing heart rate during exhalation. RSA is mainly determined by vagal influences on the heart, and therefore provides an index of parasympathetic activity (Berntson et al. 1997). Theorists suggest that resting RSA reflects the degree to which an individual is able to respond flexibly to changes in the internal and external environment (Porges 1995; Thayer and Lane 2000). They postulate that the ability of the parasympathetic nervous system to inhibit autonomic arousal during fight or flight responses to environmental challenges is essential in the regulation and expression of emotions, and they propose resting RSA as an index of these self-regulatory capacities (see also Appelhans and Luecken 2006). When confronted with a social stimulus, individuals either attend to and engage with it or perceive it as threatening and initiate fight or flight responding. Whereas social engagement requires sustained attention, characterized by increased vagal activity producing heart rate deceleration, fight or flight responses require large sympathetically mediated heart rate accelerations and decreased vagal activity. In situations of rest, RSA reflects the tonic activity of the vagal nerve. Hence, a low basal RSA, indicating reduced vagal tone, may mark a predisposition to show fight and flight responding and thus a low threshold to show aggression (Beauchaine et al. 2001; Porges 1995). Low resting RSA has also been related to a wide range of psychiatric disorders that are characterized by affect dysregulation, such as depression, anxiety and aggression (Beauchaine 2001; Thayer and Lane 2000).

### Associations between Resting RSA and Externalizing Behavior

Because low resting RSA may be related to a low threshold to show aggression, it may also be related to externalizing behavior. Research on the association between resting RSA and externalizing behavior in adolescence has mainly been conducted in clinical or high risk samples, and has often only involved boys. Negative associations were quite consistently found in clinical studies (e.g., Beauchaine et al. 2007; De Wied et al. 2009), as well as in samples consisting of boys at risk for externalizing behavior (e.g., Mezzacappa et al. 1997; Pine et al. 1998). However, results of studies that investigated this link in community samples have been inconsistent. In samples ranging from school age to young adulthood, the association between resting RSA and externalizing behavior has been found to be negative (e.g., Xu et al. 2014), negative only for boys (El-Sheikh and Hinnant 2011), non-significant (e.g., El-Sheikh and Whitson 2006), and even positive (Scarpa and Ollendick 2003). Inconsistencies in results may be due to differences in the assessment of RSA (e.g., under standardized laboratory conditions vs. assessment at schools) or sample characteristics (e.g., school age children vs. young adults).

### Associations between Resting RSA and Empathic Concern

To be able to show empathic concern, self-regulation is thought to be essential. Empathic concern, also labeled sympathy, is an emotional response stemming from the apprehension of another’s emotional state that consists of feelings of compassion for the other. When lacking adequate self-regulation, the confrontation with others’ emotions may, instead of resulting in empathic concern, bring about a self-focused, aversive, emotional reaction to the vicarious experiencing of another’s emotion (Eisenberg and Eggum 2009). Hence, as a physiological index of self-regulation, low resting RSA may be related to lower empathic concern.

Yet, results of the few studies that investigated the association between resting RSA and empathic concern are inconsistent. Positive associations have been reported among toddlers, although marginally significant (Liew et al. 2011), and among school-aged children, but in 1 study only in girls (Fabes et al. 1993) and in another study only in boys (Eisenberg et al. 1996). Further, resting RSA has been found positively related to concordance between adolescents’ own and their mothers’ affect (Diamond et al. 2012), but Oveis and colleagues (2009) found no association between young adults’ resting RSA and their responses to compassion-inducing stimuli. Thus, despite the conceptual reasons to expect higher resting RSA to predict higher empathic concern, empirical evidence is equivocal (as was the case for the association between RSA and externalizing behavior) and studies on this link in adolescence are lacking.

### The Moderating Role of Resting RSA

Besides the potential direct associations between resting RSA and social functioning, recent research suggests that RSA may also play a moderating role in the relation between environmental influences and children’s social adjustment (e.g., Eisenberg et al. 2012; El‐Sheikh 2005), although theories regarding the nature of this interaction are diverging. On the one hand, high resting RSA may function as a protective factor. Individuals with high levels of resting RSA are expected to have better self-regulatory capacities (Beauchaine 2001; Thayer and Lane 2000) and may, because of this, be better able to cope with environmental stressors. If high RSA indeed functions as a protective factor, negative environmental influences are expected to have impact on individuals low in RSA, but not (or to a lesser extent) on individuals high in RSA. On the other hand, high resting RSA may function as a susceptibility factor. According to differential susceptibility theory and the notion of biological sensitivity-to-context, certain characteristics that make individuals more vulnerable to environmental adversity also make them more likely to benefit from positive environmental influences (Belsky 1997; Boyce and Ellis 2005; Ellis et al. 2011). Individuals high in resting RSA may, because of their more active engagement with their environment (Beauchaine 2001; Thayer and Lane 2000), be more sensitive to environmental influences than adolescents low in resting RSA. This would imply stronger effects of both positive and negative environmental factors on individuals high in resting RSA than on individuals low in resting RSA.

In line with the perspective of high resting RSA as a protective factor, some studies indeed found high resting RSA to buffer the impact of adverse environmental influences on children’s social adjustment. For instance, in a sample of school aged children, the relation between parental problem drinking and children’s adjustment was stronger for children low in resting RSA than for children high in resting RSA (El‐Sheikh 2005). Also, among 9 to 16 year boys, maltreatment was positively related to aggression, but only for boys with low levels of RSA (Gordis et al. 2010). In addition, the relation between marital conflict and behavior problems was stronger for children low in resting RSA than for children high in resting RSA (Katz and Gottman 1997; El-Sheikh et al. 2011).

In line with the perspective of high resting RSA as a susceptibility factor, some studies found children high in resting RSA to be more responsive to environmental influences than children low in resting RSA. Among 4- to 7-year olds, maternal depressive symptomatology was negatively related to emotion regulation for children high in resting RSA, but not significantly related for children low in resting RSA (Blandon et al. 2008). Further, the association between parental psychiatric symptomatology and problem behavior was stronger for children high in resting RSA than for children low in resting RSA (Shannon et al. 2007). In addition, Eisenberg and colleagues (Eisenberg et al. 2012) found that environmental quality negatively predicted mother-reported aggression for toddlers with high and average resting RSA, but not for children low in RSA. However, resting RSA did not significantly moderate the association between stressful life events and adolescents’ externalizing behavior (Oldehinkel et al. 2008).

Thus, although a growing body of literature suggests RSA moderates the relation between contextual influences and children’s adjustment, both theoretical notions and results of empirical research are conflicting about the direction of the effects. Hence, we hypothesize resting RSA to play a moderating role, but our study is exploratory regarding the direction of the interaction. Moreover, whereas most studies have only investigated the effects of negative contextual influences (e.g., Oldehinkel et al. 2008; Blandon et al. 2008), or have only assessed negative outcomes (e.g., Eisenberg et al. 2012; Shannon et al. 2007), we explore the interaction effects of resting RSA with negative as well as positive environmental factors on both negative and positive outcomes, and we also test whether adolescents with varying levels of resting RSA are affected differentially by both environmental risk and benefit.

### The Current Study

The first aim of the current 2-wave longitudinal study was to examine in a community sample of adolescent boys and girls whether resting RSA at age 17 could predict externalizing behavior and empathic concern 1 year later. Although low resting RSA has conceptually been related to high externalizing behavior and studies among boys in clinical samples have indeed revealed negative associations, previous findings of the few studies in community samples have provided little evidence for this notion. Also with regard to empathic concern, previous research is scarce and does not provide clear evidence for resting RSA as an important contributor. Moreover, both theory and empirical research suggest that resting RSA may interact with environmental influences in predicting social adjustment. Therefore, the second aim was to examine whether the interaction between resting RSA and parent-adolescent relationship quality predicted relative changes in externalizing behavior and empathic concern. Individuals high in resting RSA may, due to their better self-regulation, be less affected by negative environmental influences or, conversely, they may be more sensitive to positive as well as negative influences due to their higher emotional reactivity and higher engagement with their environment. Therefore, we investigated the effects of both a positive environmental characteristic (i.e., perceived parental support) and a negative characteristic (i.e., perceived negative interaction with parents) of the parent-adolescent relationship on both adaptive behavior (i.e., empathic concern) and maladaptive behavior (i.e., externalizing behavior). Parent-adolescent relationship quality has been found to be associated with adolescents’ externalizing behavior (for a review see Branje et al. 2008) as well as with empathic concern (e.g., Miklikowska et al. 2011). We hypothesized the direction and strength of these associations to be dependent on adolescents’ levels of resting RSA, but our study is explorative with regard to the pattern of the interaction because of the diverging theories and inconsistent results of previous research on this issue. Further, based on sex differences that previous studies found in associations between resting RSA and social functioning (e.g., Eisenberg et al. 1996; Fabes et al. 1993; Gordis et al. 2010), we investigated the relations separately for boys and girls.

## Method

### Sample

This 2-year longitudinal study used data from the ongoing Research on Adolescent Development and Relationships (RADAR) project. Adolescents participating in RADAR Young (*N* = 497) were recruited from randomly selected schools in the province of Utrecht and four cities in The Netherlands. They participated in seven annual home visits during which they completed several questionnaires. The current study used data from a subsample that also participated in an individual laboratory session at the university (*n =* 382). The laboratory session took place around the time of the fifth annual questionnaire wave. In addition, we used questionnaire data of adolescents and their mothers collected during the fifth and sixth wave (from now on referred to as Time 1 and Time 2). Of all adolescents who participated in the laboratory session, data of three participants were lost due to technical problems or experimenter error, and thus the sample consisted of 379 adolescents with 212 boys (*M* age at Time 1 = 17.04, *SD* = 0.46) and 167 girls (*M* age at Time 1 = 16.94, *SD* = 0.41). The majority of the adolescents was native Dutch (95.8 %), lived with both parents (78.4 %), and came from families classified as medium or high socioeconomic status (91.8 %).

### Procedure

#### Home Visits

During the home visits, adolescents and their mothers filled out a battery of questionnaires. A trained research assistant provided verbal instructions in addition to the written instructions that accompanied the questionnaires. For each home visit, parents provided written informed consent before adolescents participated. Adolescents received 30 Euros for their participation in each of the home visits.

#### Laboratory Session

Adolescents visited the university to participate in an individual laboratory session during which (among other assessments) resting RSA was assessed. Parents and adolescents both provided written informed consent before participation of the adolescent in this session. The session took place in a testing room equipped with a personal computer and a 17-inch computer screen (HP 1730) for presentation of an aquatic video (see below). An adjacent observation room with a one-way mirror, through which the experimenter could observe the participant, was equipped with a personal computer for online monitoring of physiological data collection. Both computers were connected to a portable digital recorder for preprocessing and storage of physiological data (Vitaport III, TEMEC Instruments B.V., Kerkrade, The Netherlands), which was attached to the participant’s chair. A trained female experimenter, who followed a written protocol detailing the verbal instructions and electrode placement, received the participant. After familiarizing the participant with the procedure, electrodes were attached for ECG recording and the participant was seated in a comfortable chair at a table facing the monitor of the stimulus computer (at approximately 90 cm distance). Participants were instructed to relax and watch an aquatic video after which the experimenter dimmed the light and left the testing room. ECG was continuously recorded throughout the time the participants watched the video. Adolescents received 50 Euros for their participation in the laboratory session.

This study was approved by the Board of the local research institute and by the Medical Ethical Committee of the Utrecht Medical Centre.

## Materials

### Relaxation Video

A 5-min fragment from an aquatic video (*Coral Sea Dreaming*, Small World Music, Inc.) was presented on the computer screen. This video has been found to foster relaxation resulting in lower levels of cardiovascular activity than obtained during a traditional resting state without external stimulation (Piferi et al. 2000).

### Measures

#### Resting RSA

The electrocardiogram (ECG) was recorded with electrodes on the chest (sternum-V6 lead). The ECG signal was anti-aliasing filtered (512 Hz lowpass filter), digitized at a rate of 1024 Hz, and digitally bandpass filtered (5–30 Hz) to suppress baseline shifts, exceptionally large T-waves, and high-frequency artifacts such as EMG potentials. A computer-assisted procedure was executed to detect ECG R-waves and to make corrections for (a) prolonged heart periods due to missing R-waves and (b) short heart periods due to false R-waves (for details, see De Wied et al. 2009). RSA during the presentation of the aquatic film clip was determined by applying power spectral analysis on the heart periods using the computer program CARSPAN for Windows 1.34 (Mulder et al. 2007). The 300-s presentation period was divided into five 50 % overlapping data segments of 100 s duration consisting of a series of R-wave events as a function of time. Each 100-s data segment was cross-multiplied with a cosine window tapering 5 % of each end of the segment. Next, it was subjected to power spectral analysis using the Sparse Direct Fourier Transform algorithm (Rompelman et al. 1982) that was directly applied on the arrival times of the R-waves in the ECG without interpolation of the non-equidistant time series. Spectral power was determined in the 0.15–0.40 Hz frequency band, averaged across the five data segments, and subjected to natural logarithmic transformation. The power in this frequency band is associated with RSA and is generally believed to be an index of tonic parasympathetic control of the heart (Berntson et al. 1993; Berntson et al. 1997). This RSA measure has shown good temporal stability within experimental sessions (Sloan et al. 1995) or across sessions repeated with 1-week intervals (Bertsch et al. 2012).

#### Perceived Quality of Adolescent-Parent Relationship

The perceived quality of the relationship with parents was measured during the home visit at Time 1, using two subscales of the Network of Relationships Inventory – Short Form (NRI; Furman and Buhrmester 1985; Furman and Buhrmester 1992): support and negative interaction. Adolescents completed these subscales separately for the relationship with their father and their mother. The support subscale consists of eight items tapping relationship qualities like affection, companionship, and admiration (e.g., “how much does your mother/father really care about you?”). The negative interaction subscale consists of six items tapping the intensity of negative interaction in the relationship (e.g., “do you and your mother/father get at each other’s nerves?”). Items were rated at a 5-point Likert scale, ranging from 1 *(a little or not at all*) to 5 (*more is not possible*). Previous studies provided support for the reliability and validity of the NRI (De Goede et al. 2009; Furman and Buhrmester 1985, 1992). For the current sample, Cronbach’s alpha for perceived support from mothers and fathers was 0.85 and 0.89, respectively, and for perceived negative interaction with mothers and fathers, it was 0.95 and 0.94, respectively. The scores on the relationship with the mother and father were averaged providing a single score for perceived support from parents and a single score for perceived negative interaction with parents.

#### Externalizing Behavior

During the home visits at Time 1 and Time 2, adolescents reported on their antisocial behavior, using the subscale externalizing behavior of the Youth Self Report questionnaire (YSR; Achenbach 1991; Verhulst et al. 1996). The externalizing behavior subscale consists of 30 items assessing whether the adolescent shows aggressive behavior (e.g., “I destroy things that belong to others”) and delinquent behavior (e.g., “I lie or cheat”). Items were rated on a three-point Likert scale, ranging from 0 (*never*) to 2 (*often*). Previous studies found support for the reliability and validity of the Dutch version of the YSR (e.g., De Groot et al. 1996). For the current sample, Cronbach’s alpha on externalizing behavior was 0.88 at Time 1 and 0.88 at Time 2. The scores on externalizing behavior were logarithmically transformed to reduce skewness.

#### Empathic Concern

Adolescents’ self-reported empathic concern was measured at Time 1 and Time 2, using a seven-item subscale of the Dutch version of the Interpersonal Reactivity Index (IRI; Davis 1983; Hawk et al. 2013). This subscale assessed adolescents’ tendency to sympathize with others in need. A sample item is “I often have tender, concerned feelings for people less fortunate than me”. Adolescents scored the items on a 5-point Likert scale, ranging from 0 (*doesn’t describe me at all*) to 4 (*describes me very well*). The Dutch version of the IRI has adequate internal consistency and validity (Hawk et al. 2013). For the current sample, Cronbach’s alpha on empathic concern was 0.70 at Time 1 and 0.72 at Time 2.

### Missing Data

Adolescents who participated in the laboratory session did not significantly differ in empathic concern from their counterparts who joined the annual questionnaire waves but who did not participate in the test session and who therefore were not part of the current sample, *t* (442) = 0.93, *p* = 0.36. However, participating adolescents reported higher externalizing behavior at Time 1 than adolescents who did not participate in the current study, *t* (442) = -2.26, *p* = 0.02. Within the current sample of adolescents, across all measures on average 5.0 % (ranging from 0.0 % to 9.8 %) of the data were missing. Little’s Missing Completely at Random (MCAR) test revealed *χ* ^2^ (86) = 90.89, *p* = 0.34, and normed *χ* ^*2*^ *(χ* ^*2*^ */df)* = 1.06, indicating that the data were likely missing at random and that missing values could safely be imputed (Bollen 1989).

The Expected Maximization algorithm in the Multiple Imputation module of LISREL9.1 was used to generate 10 datasets that were combined to obtain overall estimates of the missing values (see Schafer 1997 for details on the procedure). The imputed dataset, containing 379 cases, was used in all statistical analyses.

### Statistical Analyses

First, descriptive statistics were computed for all study variables, and independent sample t tests were conducted to test for sex differences in mean levels on these variables. Second, bivariate correlations between study variables were calculated. Third, we ran a series of regression models, using robust maximum likelihood estimation, in MPlus 7.0 (Muthén and Muthén 2012). To investigate whether the Time 1 variables resting RSA, parental support, and parental negative interaction could predict externalizing behavior and empathic concern 1 year later at Time 2, a separate model was run for each of the two outcome variables. Moreover, to test whether resting RSA moderated the association between relationship quality and adolescents’ social functioning, we included in these models the effects of the 2-way interactions between resting RSA and parental support, and between resting RSA and parental negative interaction, on each outcome variable. Concurrent associations between all predictors were included in the models although for reasons of clarity only associations with the dependent variables are reported. Further, we added age of the adolescent and socioeconomic status of their families as control variables to the models. Since this did not alter our findings, results of the models without these control variables are presented. All predictor variables were standardized to a mean of 0 and a standard deviation of 1, and interaction terms were computed using these standardized variables. All models were run using a multi-group approach in which the associations were explored separately for boys and girls, and in which sex differences in these associations were tested. Models in which all parameters were constrained to be equal were compared to the baseline model in which all parameters were free to vary across gender. We used the chi-square difference test, delta RMSEA (> 0.015) and delta CFI (> 0.010) to compare model fit (Chen 2007). If the results of at least two of the three tests for model comparison indicated the constrained model to fit significantly worse than the baseline model, associations were assumed to differ between boys and girls (Kline 2005) and results of the unconstrained model are reported. In addition, for paths of interest (the paths from resting RSA, and the interaction terms including RSA, to the dependent variable) we tested whether constraining each path separately worsened the model fit significantly.

Finally, for the significant interactions we examined whether the shape of the interaction was indicative of high resting RSA as a protective factor or of high resting RSA as a susceptibility factor. The interaction was in support of resting RSA as a protective factor if the association between parent-adolescent relationship quality and adolescents’ adjustment was negative for adolescents low on resting RSA, but non-significant (or at least weaker) for those high on resting RSA. The interaction was in support of differential susceptibility if two conditions were met: (1) parent-adolescent relationship quality was significantly related to adjustment for adolescents high, but not low, on resting RSA, (2) high resting RSA was related to better adjustment for adolescents who experience a positive relation with their parents and was related to lower adjustment for adolescents who experience a negative relation with their parents (i.e., “cross-over” interaction; see Ellis et al. 2011). To examine this, simple slopes for each interaction were presented in two different ways: first, with resting RSA as the moderator variable, and second, with parent-adolescent relationship quality as the moderator variable (at 1 SD above and below mean). We applied the Johnson-Neyman technique, using the computational tool of (Preacher et al. 2006), to identify for which regions in the range of the moderator variable, effects of the focal predictor on the outcome were significant (*p* < 0.05) (Bauer and Curran 2005; Hayes and Matthes 2009).

## Results

### Preliminary Analyses

Table 1 presents means and standard deviations of all study variables separately for boys and girls. Independent sample *t* tests revealed a sex difference in adolescents’ resting RSA. Girls showed higher levels of resting RSA than boys. Further, girls reported higher levels of support from their parents, and higher levels of empathic concern than boys at both Time 1 and Time 2. We found no significant sex differences in perceived negative interaction with parents, nor in externalizing behavior at Time 1 and Time 2.

**Table 1:** Mean scores for boys and girls on resting RSA, parental support, negative interaction with parents, externalizing behavior and empathic concern

	Total (*N* = 379)	Boys (*n* = 212)	Girls (*n* = 167)	Boys vs. Girls (*df* = 377)	
	*M* (SD)	*M* (SD)	*M* (SD)	*t*	*η* ^*2*^
Resting RSA					
Time 1	7.61 (0.89)	7.47 (0.91)	7.79 (0.84)	-3.62***	0.04
Parental support					
Time 1	3.47 (0.58)	3.42 (0.55)	3.55 (0.60)	-2.26*	0.01
Negative interaction with parents					
Time 1	1.79 (0.56)	1.77 (0.57)	1.82 (0.56)	-0.93	0.002
Externalizing behavior					
Time 1	0.28 (0.17)	0.29 (0.18)	0.26 (0.17)	1.72	0.01
Time 2	0.26 (0.17)	0.27 (0.17)	0.25 (0.17)	1.16	0.003
Empathic concern					
Time 1	2.45 (0.56)	2.24 (0.53)	2.71 (0.50)	-8.76***	0.17
Time 2	2.46 (0.58)	2.26 (0.55)	2.73 (0.52)	-8.45***	0.16

* *p* < 0.05 *** *p* < 0.001

Bivariate correlations for boys and girls in Table 2 showed that boys with higher levels of resting RSA reported higher empathic concern at Time 1. Boys’ resting RSA was not significantly related to parental support, negative interaction with parents, empathic concern at Time 2, and externalizing behavior at Time 1 and Time 2. Girls higher resting RSA was related to higher support and lower negative interaction with parents. Girls’ resting RSA was not significantly related to empathic concern at Time 1 and Time 2, and to externalizing behavior at Time 1 and Time 2. Further, with regard to the associations between parent-adolescent relationship quality and the outcome measures, for boys higher parental support was significantly related to higher empathic concern at both Time 1 and Time 2, and to lower externalizing behavior at Time 1, but not at Time 2. Boys’ negative interaction with parents correlated positively to externalizing behavior at Time 1 and Time 2, but was not significantly related to empathic concern at Time 1 and Time 2. For girls, higher parental support was at both Time 1 and Time 2 related to lower externalizing behavior and higher empathic concern. Girls’ higher negative interaction with parents was related to lower empathic concern and higher externalizing behavior at Time 1 and Time 2.

**Table 2:** Intercorrelations of resting RSA, support from parents, negative interaction with parents, externalizing behavior, and empathic concern for boys (below diagonal) and girls (above diagonal)

	1.	2.	3.	4.	5.	6.	7.
1. Resting RSA (Time 1)	--	0.17*	-0.17*	0.00	0.01	0.04	0.00
2. Support from parents (Time 1)	0.04	--	-0.48***	-0.24**	-0.23**	0.23**	0.21**
3. Negative interaction with parents (Time 1)	0.03	-0.26***	--	0.49***	0.30***	-0.22**	-0.16*
4. Externalizing behavior (Time 1)	-0.07	-0.21**	0.39***	--	0.76***	-0.19*	-0.20**
5. Externalizing behavior (Time 2)	-0.09	0.03	0.35***	0.71***	--	-0.24**	-0.31***
6. Empathic concern (Time 1)	0.14*	0.29***	-0.13	-0.28***	-0.15*	--	0.58***
7. Empathic concern (Time 2)	-0.02	0.24***	-0.11	-0.20**	-0.12	0.66***	--

* *p* < 0.05 ** p < 0.01 *** *p* < 0.001

### Regression Models

#### Externalizing Behavior

**Fig. 1:**
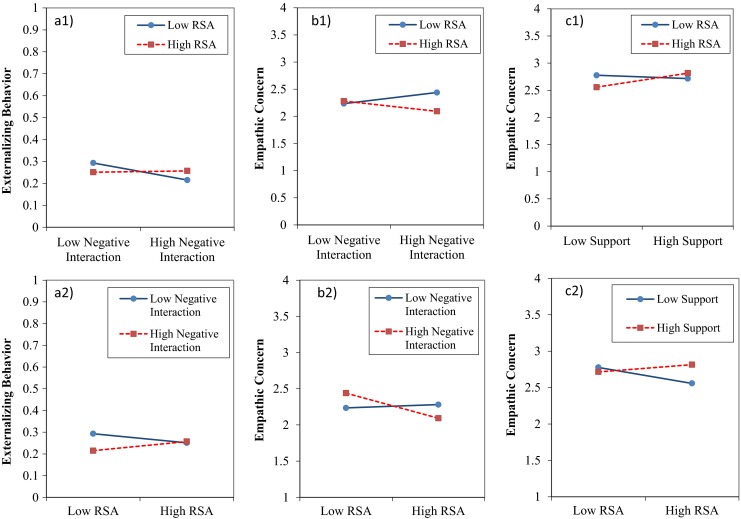
**a** Interaction between girls’ perceived negative interaction with parents and RSA, predicting (the logarithm of) externalizing behavior, **b** Interaction between boys’ perceived negative interaction with parents and RSA predicting empathic concern, **c** Interaction between girls’ perceived support from parents and RSA predicting empathic concern. RSA at 1 SD above and 1 SD below mean. Figures a1 to c1 present the interaction effects with RSA as the moderator variable. Figures a2 to c2 present these same interactions, but with, respectively, negative interaction with parents and parental support as the moderator variable

A regression model was run to predict externalizing behavior at Time 1 and Time 2, with resting RSA, parental support, negative interaction with parents, and the interactions between resting RSA and parental support and between resting RSA and negative interaction with parents as predictors. Multiple Group analyses revealed the constrained model to fit significantly worse than did the model in which paths were free to vary between boys and girls, Δχ2 (6) = 19.379, *p* < 0.001, ΔRMSEA = 0.108, ΔCFI = 0.064. Therefore, results of the unconstrained model are reported (see Table 3). With regard to longitudinal associations, externalizing behavior showed considerable stability over time for both boys and girls. For boys, a higher negative interaction with parents at Time 1 predicted higher externalizing behavior at Time 2. Remarkably, boys who perceived their parents as more supportive, reported more externalizing behavior 1 year later. However, this may be a suppression effect, given that the correlation matrix showed the correlation to be non-significant. Boys’ resting RSA did not significantly predict externalizing behavior at Time 2, and also the interaction effects were not significant. For girls, there were no significant longitudinal main effects of resting RSA, parental support or negative interaction with parents, but the interaction between resting RSA and negative interaction with parents significantly predicted girls’ externalizing behavior 1 year later. Probing the interaction, revealed that for girls with resting RSA levels ≤ 0.22 *SD* below mean, higher negative interaction with parents was related to lower externalizing behavior (*b* = -0.02). For higher levels of resting RSA, the association did not become significant within the range of scores of the current sample. Probing this interaction for varying levels of girls’ negative interaction with their parents revealed that resting RSA was positively related to externalizing behavior for girls who reported negative interaction with parents at levels ≥ 1.39 *SD* above mean (*b* = 0.03), whereas the association was negative for girls who reported negative interaction with parents at levels ≤ 1.19 *SD* below mean (*b* = 0.01). The interaction effects are visualized in Fig. 1 by showing simple slopes for girls high and low in resting RSA in Fig. 1.a1, and for girls high and low in negative interaction with parents in Fig. 1.a2. It should be noted that constraining the interaction path to be equal between boys and girls did not significantly worsen the model fit, Δχ2 (1) = 1.548, *p* = 0.21, ΔRMSEA = 0.054, ΔCFI = 0.003, indicating that the strength or direction of this path did not differ significantly between boys and girls.

**Table 3:** Concurrent and longitudinal associations of boys’ and girls’ resting RSA, perceived parental support and negative interaction with externalizing behavior and empathic concern

	Externalizing behavior				Empathic concern			
	Boys		Girls		Boys		Girls	
	*β*	b (SE)	*β*	b (SE)	*β*	b (SE)	*β*	b (SE)
*Concurrent Effects t1 → t1*								
Resting RSA	-0.07	-0.07 (0.06)	0.01	0.00 (0.08)	0.14+	0.14 (0.07)	0.04	0.04 (0.08)
Support from parents	-0.21**	-0.21 (0.09)	-0.24**	-0.24 (0.09)	0.29***	0.29 (0.07)	0.23**	0.22 (0.08)
Negative interaction with parents	0.39***	0.39 (0.08)	0.49***	0.49 (0.09)	-0.13+	-0.13 (0.08)	-0.22**	-0.22 (0.08)
RSA X support	-0.04	-0.04 (0.08)	0.09	0.08 (0.07)	-0.02	-0.02 (0.08)	-0.05	-0.05 (0.08)
RSA X negative interaction	0.06	0.06 (0.15)	-0.06	-0.06 (0.09)	0.11	0.10 (0.08)	0.13+	0.13 (0.09)
*Stability Effects t1 → t2*								
Externalizing behavior	0.70***	0.12 (0.01)	0.79***	0.14 (0.01)	-	-	-	-
Empathic concern	-	-	-	-	0.68***	0.37 (0.04)	0.57***	0.29 (0.04)
*Longitudinal Main Effects t1 → t2*								
Resting RSA	-0.06	-0.01 (0.01)	-0.00	0.00 (0.01)	-0.14*	-0.08 (0.03)	-0.06	-0.03 (0.03)
Support from parents	0.22**	0.04 (0.01)	-0.09	-0.02 (0.01)	0.06	0.03 (0.03)	0.10	0.05 (0.05)
Negative interaction with parents	0.13*	0.02 (0.01)	-0.10	-0.02 (0.01)	0.01	0.00 (0.03)	0.00	0.00 (0.04)
*Longitudinal Interaction Effects t1 → t2*								
RSA X support	-0.04	-0.01 (0.01)	0.01	0.00 (0.01)	-0.06	-0.03 (0.03)	0.15*	0.08 (0.04)
RSA X negative interaction	0.01	0.00 (0.01)	0.13*	0.02 (0.01)	-0.17**	-0.10 (0.03)	0.04	0.02 (0.03)
	*R* ^*2*^		*R* ^*2*^		*R* ^*2*^		*R* ^*2*^	
	0.55		0.60		0.47		0.37	

+ *p* < 0.10 * *p* < 0.05 ** p < 0.01 *** *p* < 0.001

#### Empathic Concern

Table 3 summarizes the results of the regression model predicting empathic concern at Time 2 with EC at Time 1, resting RSA, parental support, negative interaction with parents, and the interactions between resting RSA and parental support and between resting RSA and negative interaction with parents as predictors. Multiple Group analyses revealed the constrained model to fit significantly worse than did the model in which paths were free to vary between boys and girls, Δχ2 (6) = 11.50, *p* = 0.07, ΔRMSEA = 0.07, ΔCFI = 0.033. Therefore, results of the unconstrained model are reported (see Table 3).

With regard to the longitudinal associations, empathic concern showed considerable stability over time for both boys and girls. The only significant main effect was boys’ higher resting RSA at Time 1 predicting lower empathic concern at Time 2, which was qualified by a significant interaction effect. The interaction between resting RSA and perceived negative interaction with parents at Time 1 significantly predicted boys’ empathic concern at Time 2, indicating that the association between negative interaction and empathic concern differs for boys with varying levels of resting RSA. This interaction was not significant for girls. Further, constraining this path to be equal between boys and girls significantly worsened the model fit, Δχ2 (1) = 8.72, *p* < 0.001, ΔRMSEA = 0.20, ΔCFI = 0.067, suggesting that the strength of the interactive effect of RSA and negative interaction on empathic concern differed significantly between boys and girls. Probing the interaction, revealed that for boys with resting RSA levels ≥ 0.83 *SD* above mean, higher negative interaction with parents significantly predicted lower empathic concern (*b* = -0.08). Remarkably, the reverse was true for boys with resting RSA levels ≤ 0.73 *SD* below mean: their higher negative interaction with parents significantly predicted higher empathic concern (*b* = 0.08). Probing this interaction for varying levels of boys’ negative interaction with parents revealed that resting RSA was negatively related to empathic concern for boys who reported negative interaction with their parents at levels ≥ 0.16 *SD* below mean (*b* = 0.03). For lower levels of negative interaction with parents, the association did not become significant within the range of scores of the current sample. The interactive effects are visualized by showing simple slopes for boys high and low in resting RSA in Fig. 1.b1, and for boys high and low in negative interaction with parents in Fig. 1.b2.

Interestingly, for girls (but not for boys), the interaction between resting RSA and perceived support from parents at Time 1 significantly predicted empathic concern at Time 2. Thus, the association between parental support and empathic concern differed for girls varying in level of resting RSA. Further, constraining this path to be equal between boys and girls significantly worsened the model fit, Δχ2 (1) = 10.76, *p* = 0.00, ΔRMSEA = 0.23, ΔCFI = 0.059, indicating that the strength of the interactive effect differed significantly between boys and girls. Probing the interaction revealed that for girls with resting RSA levels ≥ 1.34 *SD* above mean, higher support from parents significantly predicted higher empathic concern (*b* = 0.16). For girls with low resting RSA levels there was no significant association between parental support at Time 1 and empathic concern at Time 2, within the range of RSA scores in the current sample. Probing this interaction for varying levels of parental support revealed that resting RSA was negatively related to empathic concern for girls who reported parental support at levels ≤ 0.61 *SD* below mean. For higher levels of parental support, the association did not become significant within the range of scores of the current sample. The simple slopes for girls with high and low resting RSA are depicted in Fig. 1.c1, and the simple slopes for girls with high and low perceived parental support are depicted in Fig. 1.c2.

## Discussion

Our first aim was to examine whether resting RSA predicted adolescents’ empathic concern and externalizing behavior. Our second aim was to investigate whether and how adolescents’ resting RSA moderated the associations between parent-adolescent relationship quality and, respectively, adolescents’ empathic concern and externalizing behavior. We examined whether the interaction patterns were consistent with either the notion that high resting RSA functions as a protective factor (Ellis et al. 2011), or the notion that high resting RSA functions as a susceptibility factor (Beauchaine 2001; Thayer and Lane 2000). Whereas previous studies have almost exclusively focused on the effects of environmental adversity on children’s dysfunction, we explored the interaction effects of resting RSA with both negative and positive environmental factors on both negative and positive outcomes. Moreover, we investigated not only whether adolescents high in resting RSA were more susceptible to environmental influences than adolescents low in resting RSA, but we also tested whether they were stronger affected by both environmental adversity and benefit.

Despite conceptual reasons to expect resting RSA to be negatively linked to externalizing behavior and positively linked to empathic concern, our findings did not support this. With regard to externalizing behavior, we found no main effects of boys’ and girls’ resting RSA. Our finding is in contrast with results in clinical samples (e.g., Beauchaine et al. 2007; Mezzacappa et al. 1997), but is in line with several studies conducted in community samples that also did not find a significant association between resting RSA and externalizing behavior (e.g., Calkins et al. 2007; El-Sheikh and Whitson 2006). This suggests that low basal RSA is a marker of dysregulation for youth showing externalizing behavior in the clinical range rather than for relatively well-functioning adolescents. In a community sample of adolescents, certain levels of externalizing behavior are part of the normative development instead of an expression of pathological dysregulation (Moffitt 1993). Also with regard to empathic concern, our results did not support the expectation that high resting RSA would be a positive predictor (e.g., Fabes et al. 1993). Only for boys, we concurrently found a tendency towards a positive correlation, but the longitudinal analyses revealed the inverse association (which was qualified by a significant interaction, interpreted below). Thus, our findings as well as the inconsistent results of previous studies in community samples, suggest that above a certain threshold inter-individual differences in resting RSA may have less impact on social functioning than at lower levels. Future research may test this by comparing adolescents with scores on problem behavior in the clinical range with adolescents who score within the normal range. Further, a relationship between biological factors and problem behavior may emerge rather in interaction with environmental risk factors than as a direct association (for reviews see Raine 2005; Moffitt 2005).

Our findings did reveal support for resting RSA as a moderator in the association between parent-adolescent relationship quality and adolescents’ adjustment. For boys, resting RSA interacted with negative interaction in the prediction of empathic concern. For girls, resting RSA interacted with negative parent-adolescent interaction in the prediction of externalizing behavior, and with parental support in the prediction of empathic concern.

Looking across the interaction patterns, no support was found for high resting RSA as a buffer for the impact of low environmental quality; the effects of high negative interaction with parents or low parental support were not stronger for adolescents with low RSA than for adolescents with high RSA. In fact, the most consistent finding was that adolescents high in resting RSA appeared to be hampered relatively more by a negative environment than were adolescents low in resting RSA. That is, boys high in RSA reported less empathic concern if they experienced higher negative interaction with their parents, and girls high in RSA reported less empathic concern if they experienced lower parental support. This finding is consistent with previous studies that also found, only for children high in RSA, the association between environmental quality and social adjustment to be negative (Eisenberg et al. 2012; Blandon et al. 2008; Shannon et al. 2007). The fact that our findings were not in line with the notion that individuals with high RSA levels are better able to cope with environmental stressors, may be explained by the restricted range of scores on the relationship quality measures in this relatively low risk sample. Low scores may not reflect a truly adverse environment. Perhaps because of better attentional skills (Thayer and Lane 2000), adolescents with high resting RSA are more aware of relatively small negative changes in the relationship with their parents than are adolescents with low resting RSA.

Yet, the interaction patterns were also not clearly in line with the notion of differential susceptibility. According to the first necessary condition for establishing differential susceptibility, girls high in resting RSA indeed appeared to be more susceptible to parental support than girls low in resting RSA. That is, parental support was positively related to empathic concern for girls high in resting RSA, whereas the association was non-significant for girls low in resting RSA. Yet, the interaction pattern did not meet the second necessary condition, that is, that high susceptible individuals should be stronger affected both by positive and negative factors. Girls who were higher in resting RSA reported lower empathic concern if they experienced low parental support, but they did not report higher empathic concern if they experienced high parental support. Thus, girls high in resting RSA are, compared to girls low in resting RSA, not more susceptible to parental support for both ‘better and worse’, but only for the adverse effects of low parental support. In general, our results suggest that the negative rather than the positive end of the spectrum makes the difference (i.e., low support, high negative interaction; see Fig. 1a2 to c2). In the current sample, the average level of parenting may already be “good enough” for adolescents to be well-adjusted, and therefore differences above this level may have little effect.

The interactions between boys’ and girls’ resting RSA and negative interaction with parents showed a remarkable pattern. That is, whereas the association between boys’ negative interaction with parents and empathic was non-significant for boys high in RSA, boys with low levels of RSA reported higher levels of empathic concern if they experienced more negative interaction with their parents. Similarly, whereas girls high in RSA reported more externalizing behavior if they experienced more negative interaction with parents, girls low in resting RSA reported less externalizing behavior if they experienced more negative interaction with their parents. Thus, instead of being less responsive than adolescents high in resting RSA, as expected based on differential susceptibility theory, adolescents low in resting RSA tended to be reversely affected by negative interaction with their parents. An explanation for this finding may be that children with low resting RSA are less easy to socialize than children with high resting RSA because of their difficulties in self-regulation. As suggested by Kochanska (1991), children with certain characteristics need more forceful parental strategies in order to develop conscience. In this way, parents’ efforts to discipline and monitor these adolescents may go together with negative interaction.

Significant gender effects were found in the prediction of empathic concern. For girls (but not boys) high in resting RSA, parental support predicted empathic concern. A possible explanation is that girls generally experience more support in their relationships and exhibit a stronger relational orientation during adolescence than do boys (e.g., De Goede et al. 2009). Girls may therefore be more attuned to and affected by changes in parental support than are boys. The finding that only for boys, the interaction between resting RSA and negative interaction predicted empathic concern, is puzzling and needs further research.

Our results should be interpreted in light of some limitations. First, self-reports were used to assess parent-adolescent relationship quality, externalizing behavior and empathic concern. Especially our finding of no significant association between resting RSA and externalizing behavior may be due to adolescents’ underreports of externalizing behavior. However, previous studies have found adolescents to be more reliable reporters of their own problem behavior than parents (Edelbrock et al. 1985; Verhulst and Van der Ende 1992). Second, due to our broad measure of externalizing behavior, it is unclear whether resting RSA, though not being associated with externalizing behavior in general, may be negatively related only to specific forms of externalizing behavior (e.g., with reactive, but not proactive aggression; Scarpa et al. 2010; Xu et al. 2014). Third, resting RSA was assessed during a laboratory session several weeks apart from the home visit during which the Time 1 questionnaires were assessed. Differences in the period in between the laboratory session and the home visit may have confounded the concurrent associations. However, resting RSA has been found to be quite stable across time (e.g., El‐Sheikh 2004). Fourth, resting RSA was assessed at the beginning of the laboratory session whereas later during this session participants had to perform a public speech task which they were encouraged to prepare before at home. In an earlier study performed in a community sample of adolescents, moderate anticipatory physiological stress responses were demonstrated preceding this task (Westenberg et al. 2009). This may have influenced the assessment of resting RSA in our study although participants watched an aquatic video which has been proven to be effective in lowering cardiovascular activity levels (Piferi et al. 2000).

By investigating in a community sample the interaction of resting RSA with both positive and negative facets of the parent-adolescent relationship, the current study extended previous research that mainly has been done in clinical or at risk samples and has primarily focused on negative contextual influences. In contrast to results of clinical studies, resting RSA was not predictive of externalizing behavior or empathic concern. However, we found resting RSA to interact with parent-adolescent relationship quality. Our findings revealed no clear support for resting RSA either as a protective factor or a susceptibility factor, but provide initial evidence for the notion that, also beyond childhood, parental efforts may differentially affect individuals with varying levels of resting RSA. The marked differences in the interaction patterns across gender, and across the full scope from negative to positive environmental factors and outcomes, highlight the importance of applying such a thorough approach. The current study shows that adolescents may respond differently to parental socialization depending on their resting RSA level, but calls for future research to disentangle the patterns in parent-adolescent interaction that may explain this differential reactivity.
